# Air fryer ownership, household food-related task sharing, cooking skills, and mediterranean diet adherence among cohabiting adults in Türkiye: A cross-sectional study

**DOI:** 10.1371/journal.pone.0352090

**Published:** 2026-07-08

**Authors:** Zeynep Begüm Kalyoncu Atasoy, Eylem Coşkun, Sinem Çekirdekoğlu, Saimenur Durmaz, Halit Tanju Besler

**Affiliations:** 1 Department of Nutrition and Dietetics, Gülhane Faculty of Health Sciences, University of Health Sciences, Ankara, Türkiye; 2 Department of Nutrition and Dietetics, Faculty of Health Sciences, Istanbul Aydin University Faculty of Health Sciences, Istanbul, Türkiye; University of Petra (UOP), JORDAN

## Abstract

Air fryers are increasingly used in home kitchens, yet their relationships with domestic culinary labour and diet quality remain unclear. This cross-sectional study included cohabiting adults in Istanbul, Türkiye, with one respondent recruited per household (N = 384; 50% women); 192 participants (50%) reported owning an air fryer. Cooking and food preparation skills were assessed using a validated 33-item scale (14-item cooking skills and 19-item food skills subscales), and Mediterranean diet adherence was assessed using MEDAS. Group comparisons and robust regression models were performed. Compared with non-owners, owners had longer education duration (median 14 vs 12 years) and lower BMI (median 25.7 vs 27.4 kg/m²) (both p < 0.001). Women scored higher than men on cooking skills, food skills, and MEDAS within both ownership strata (all p < 0.01). In robust regression models, air fryer ownership was positively associated with cooking skills (β = 4.253, p = 0.028) and food skills (β = 6.589, p = 0.004). Higher MEDAS scores were associated with older age (β = 0.049 per year, p < 0.001) and regular exercise (β = 1.873, p < 0.001), whereas male sex (β=−1.311, p < 0.001), lower perceived income, and regular smoking were associated with lower MEDAS scores (p < 0.05). Air fryer owners also more frequently reported equal sharing with a spouse for selected routine kitchen tasks, particularly table setting, meal preparation, and dishwashing. Air fryer ownership was associated with higher cooking- and food-skill scores and with more equal sharing of some kitchen tasks, but not with Mediterranean diet adherence.

## Introduction

Air fryers are compact appliances that generate a “fried-like” crust primarily through forced convection of hot air, typically with little added oil. Air frying has been shown to reduce oil uptake while maintaining familiar sensory characteristics; however, the resulting nutritional profile depends largely on the foods prepared and which cooking practices it replaces, rather than on the device itself [1,2]. Accordingly, from a public health nutrition perspective air fryer adoption is best conceptualized as a marker of household food-preparation practices. It reflects how meals are produced at home, including cooking frequency, reliance on convenience products, and time-saving strategies, rather than serving as a nutritional exposure with uniform effects independent of context [3–5].

Observational studies consistently link more frequent home cooking with dietary patterns that, on average, show higher overall diet quality and lower intake of some convenience meal categories [3,4]. Yet, cooking frequency alone may not fully capture the quality of household food practices. Beyond frequency, cooking skills and food preparation confidence have been consistently associated with higher diet quality and lower consumption of ultra-processed foods (UPFs). Air fryers may also support preparation of minimally processed foods, but they are also compatible with ready-to-cook packaged products; therefore, any relationship with diet quality should be evaluated empirically rather than assumed [6–8].

Beyond diet, household cooking technologies also intersect with social organization inside the home, particularly the division of unpaid domestic labour. Across settings, cooking and kitchen-related tasks remain strongly gendered, even as men’s participation has increased over time in some contexts [4,9]. A central question in gender research is whether labour-saving household technologies are associated with more equitable divisions of unpaid work, or whether they primarily leave underlying norms and expectations intact. Time-use research suggests that domestic technologies do not necessarily translate into proportional reductions in women’s routine housework and may instead reshape standards and expectations in ways that preserve inequality [10]. Historical evidence suggests that diffusion of household appliances can alter time allocation, although observed effects depend strongly on social and economic context rather than on appliance characteristics alone [11].

Empirically, whether domestic technologies translate into more equitable household labour arrangements remains uncertain for contemporary kitchen devices. A device perceived as reducing technical complexity by offering standardized heating and requiring less active monitoring may be associated with greater participation among partners who cook less often. However, responsibilities such as meal planning, grocery shopping, and cleaning may remain concentrated in one person, thereby sustaining the mental load and unequal accountability for household foodwork [9,10,12]. Importantly, these appliances are typically designed according to general human factors and anthropometric principles intended to fit a broad range of adult users, rather than being tailored to a specific sex or “female anatomy” [13]. Nevertheless, who uses such devices in everyday life is shaped less by ergonomic feasibility and more by social norms, role expectations, and household bargaining. Evidence from Türkiye similarly documents persistent gender asymmetries in domestic task participation and time allocation, and recent work emphasizes that inequality concerns not only who performs tasks but also who carries the organizing and anticipatory burden [9,12]. In this context, evidence is limited on whether ownership of contemporary, convenience-oriented cooking devices such as air fryers is associated with more egalitarian sharing of kitchen work within couples.

The organization of kitchen work and the distribution of culinary skills within households can influence diet quality through their effects on meal preparation patterns, routine ingredient choices, and reliance on minimally processed foods. In this respect, the Mediterranean dietary pattern is a useful framework because it emphasizes plant foods, legumes, whole grains, olive oil, and fish, and it is supported by substantial evidence from observational research and clinical trials for cardiometabolic health [14]. Since Mediterranean diet adherence emphasizes minimally processed foods, higher consumption of ultra-processed foods may represent a competing dietary orientation. Adherence to this pattern often depends on practical food-related abilities such as meal planning, routine procurement of core ingredients, and basic cooking skills, which are embedded in household routines and the division of culinary responsibilities [3–5,15,16]. Air fryers may intersect with this pattern in more than one way. They may be associated with adherence if they are used for quick preparation of minimally processed foods, including vegetables, legumes, and fish, with limited added fat and reduced technical demands. Conversely, the same convenience features may increase the routine use of ready-to-cook packaged foods that are designed for appliance-based preparation, potentially displacing time-intensive Mediterranean-style meal preparation in some households [5–8]. These competing pathways suggest that the relationship between air-fryer ownership and Mediterranean diet adherence is not self-evident and should be evaluated empirically.

More broadly, air fryer ownership can be understood not as a direct nutritional exposure or as an inherently health-promoting technology, but as a household-level marker of contemporary kitchen technology adoption. It may reflect overlapping dimensions of convenience-oriented food preparation, socioeconomic positioning and purchasing capacity, interest in cooking technologies and home food preparation, household organization of culinary labour, and broader lifestyle or health-behavior patterns. From this perspective, ownership may be embedded in household routines through which visible cooking-related tasks are shared, cooking and food preparation skills are expressed, and dietary choices are made. The conceptual rationale for examining task sharing, cooking and food preparation skills, and Mediterranean diet adherence together is therefore that these domains represent interrelated components of domestic food practices.

Therefore, a cross-sectional study was conducted among adults living as cohabiting couples to examine whether air fryer ownership, conceptualized as a marker of household kitchen technology adoption and convenience-oriented food practices, was associated with more egalitarian sharing of household kitchen work, cooking and food preparation skills, and Mediterranean diet adherence. This approach aimed to situate air fryer ownership within broader domestic food-practice patterns rather than to evaluate the appliance as a causal determinant of diet quality, skills, or household labour division.

## Materials and methods

### Study design and participants

A cross-sectional study was conducted in Istanbul, Türkiye, between March and September 2025. Adults aged 18 years or older who were living as part of a cohabiting couple in the same household were eligible. To avoid duplicate reporting of shared household practices, one respondent per household was included. Participants were recruited using snowball sampling after providing written informed consent. Because participants were recruited through snowball sampling, the sample should not be considered statistically representative of all cohabiting adults in Istanbul or Türkiye. Individuals unable to complete the questionnaire due to severe communication or neurodevelopmental conditions (e.g., aphasia, dysarthria, apraxia, autism spectrum disorder) or severe psychological/mental health problems were excluded. Sample size was calculated using the finite population correction based on Turkish Statistical Institute marriage statistics for Istanbul (2019–2023), including 106,588 marriages in 2023. Assuming a 95% confidence level, 5% margin of error, and maximum variability (p = 0.50), the minimum required sample was 384, which was reached. The study received approval from the Istanbul Aydin University Non-Interventional Clinical Research Ethics Committee (Decision No. 134/2024). All participants were adults and provided written informed consent before data collection. No minors were included in the study.

### Measures and data collection

Data were collected using a structured questionnaire comprising a researcher-developed sociodemographic form, items adapted from national surveys, and validated scales. The sociodemographic form captured participant characteristics including sex, age group, education level, employment status, and socioeconomic level, along with self-reported food preparation and cooking skill level and contextual information related to household culinary practices.

Selected questionnaire items were drawn from national surveys. Sociodemographic and health-related items were adapted from the Türkiye Nutrition and Health Survey (TNHS) 2017 [17]. Measures of exercise/physical activity, tobacco use, and alcohol consumption were adapted from the Türkiye Demographic and Health Survey (TDHS) 2013 [18], and additional health- and cooking-related items were adapted from TDHS 2018 [19]. Household kitchen labour was assessed using items from the TDHS 2018 Women’s Status module, asking who typically performed meal preparation, setting and clearing the table, and washing dishes or loading the dishwasher. Responses were recorded on a categorical scale indicating the responsible person (self only, mostly self, shared equally with spouse/partner, mostly spouse/partner, spouse/partner only, other women in the household, other men in the household, paid worker, other, or no one) and were used to describe the household distribution of kitchen work and related gender-role patterns.

Cooking-related competencies were assessed using the Cooking and Food Preparation Skills Scale, originally developed by Dean and colleagues [5] and later adapted and psychometrically evaluated for Turkish populations by Keleş and Akçil Ok [20]. The instrument comprises 33 items across two subscales: cooking skills (14 items) and food preparation skills (19 items).

**Fig 1:**
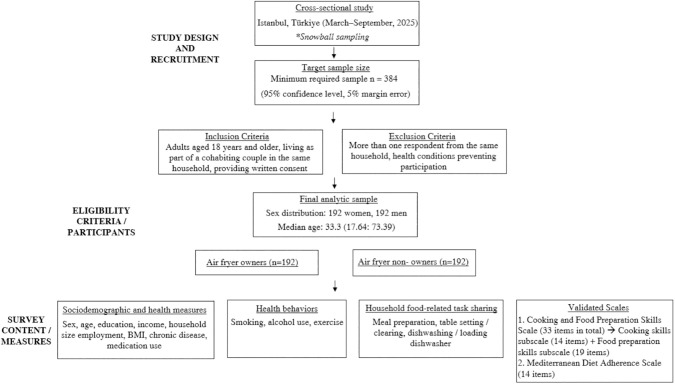
Study flow diagram of participant inclusion, survey measures, and air fryer ownership groups.

Mediterranean diet adherence was assessed using the 14-item Mediterranean Diet Adherence Screener (MEDAS), initially used in the PREDIMED trial and subsequently validated in related work, and later adapted into Turkish [16,21]. Each item is scored dichotomously (0/1) based on reported consumption, and item scores are summed to derive a total MEDAS score. In line with established cut-offs, a total score ≥7 indicates acceptable adherence and a score ≥9 indicates high/strict adherence. The overall study design, eligibility criteria, final analytic sample, ownership groups, and survey measures are summarized in Fig 1.

### Statistical analysis

Normality was assessed with the Kolmogorov–Smirnov test. Continuous variables are presented as mean ± standard deviation for approximately normally distributed data and as median (minimum–maximum) for non-normally distributed data; categorical variables are presented as frequency (percentage). For two-group comparisons of non-normally distributed continuous variables, the Mann–Whitney U test was used. Associations between categorical variables were examined using Pearson’s chi-square test and Fisher’s exact test with Monte Carlo correction, as appropriate. Where multiple category comparisons were performed, post hoc comparisons were conducted using z tests with Bonferroni adjustment. To evaluate the effects of independent variables on a non-normally distributed dependent variable, robust regression analyses were performed using the MASS package in R. Statistical significance was set at p < 0.05. Data were analyzed using IBM SPSS Statistics (version 23) and R (version 4.4.1).

## Results

### Participant characteristics by air-fryer ownership

A total of 384 adults were included (air-fryer owners: n = 192; non-owners: n = 192). Participant characteristics were compared between air-fryer owners and non-owners in Table 1. Sex distribution did not differ by air-fryer ownership (49.5% vs 50.5% men; p = 0.838). Median age was slightly lower among owners (32.35 [18.66–70.97]) than non-owners (34.30 [17.64–73.39]), but this difference did not reach conventional significance (p = 0.059). Owners reported a longer total education duration than non-owners (median 14 [3–25] years vs 12 [0–19] years; p < 0.001). Categorical education level also differed by air fryer ownership (p = 0.030), with higher education being more frequent among owners than non-owners. Overall, 51.3% of participants had higher education and 26.8% had high school education. Owners also had a lower BMI than non-owners (median 25.7 [17.21–42.3] vs 27.39 [17.21–41.49]; p < 0.001). Household size was similar in median terms (3 persons in both groups), but differed statistically by rank distribution (p = 0.024). Income status differed by ownership (p = 0.022), with fewer owners reporting financial strain (‘barely making it to the end of the month’: 15.0% vs 25.8%). Employment status also differed (p = 0.041) as owners more often reported working in the private sector (45.9% vs 33.2%), whereas non-owners more often reported being wage-earning employees in manual occupations (9.5% vs 3.6%). Chronic disease prevalence did not differ (p = 0.152), but medication use was higher among non-owners (25.3% vs 17.0%; p = 0.048). Smoking status did not differ (p = 0.960). Alcohol use differed (p = 0.043), with non-owners more frequently reporting no alcohol use (69.5% vs 57.7%) and owners more frequently reporting occasional use (36.6% vs 24.7%). Exercise status also differed (p = 0.004), with regular exercise being more common among non-owners (17.9% vs 6.2%).

**Table 1:** Participant characteristics according to air fryer ownership status (n = 384).

	Air fryer ownership status		Total	Test statistics	p-value
	Yes	No			
**Sex**					
Female	98 (50.5%)	94 (49.5%)	192 (50%)	0.042	0.838^x^
Male	96 (49.5%)	96 (50.5%)	192 (50%)		
**Age (years)**	32,35 (18.66-70.97)	34.3 (18-73.39)	33.3 (17.64: 73.39)	−1.891	0.059^z^
**Age group**					
<25 years	16 (8.2)	22 (11.6)	38 (9.9)	12.155	**0.006** ^**y**^
25–34 years	105 (54.1)^a^	76 (40)^b^	181 (47.1)		
35–44 years	39 (20.1)	34 (17.9)	73 (19)		
≥45 years	34 (17.5)^a^	58 (30.5)^b^	92 (24)		
**Total years of education**	14 (3-25)	12 (0-19)	12 (0: 25)	−3.335	**<0.001** ^**z**^
**Income Status**				9.162	**0.022** ^**y**^
More than adequate to comfortably cover monthly expenses	62 (32%)	61 (32.1%)	123 (32%)		
Adequate to meet monthly needs without difficulty	95 (49%)	77 (40.5%)	172 (44.8%)		
Sufficient for basic needs, but financial strain is present	29 (15%)^a^	49 (25.8%)^b^	78 (20.3%)		
Insufficient to meet basic needs	8 (4.1%)	3 (1.6%)	11 (2.9%)		
**Age at first marriage**	24 (12-40)	23 (15-36)	24 (12: 40)	−1.184	0.237^z^
**Employment status**					
Public-sector employee	16 (8.3%)	18 (9.5%)	34 (8.9%)	14.992	**0.041** ^**y**^
Private-sector employee	89 (45.9%)^a^	63 (33.2%)^b^	152 (39.6%)		
Self-employed	25 (12.9%)	21 (11.1%)	46 (12%)		
Student	0 (0%)	3 (1.6%)	3 (0.8%)		
Homemaker	42 (21.7%)	51 (26.8%)	93 (24.2%)		
Retired	10 (5.1%)	9 (4.7%)	19 (5%)		
Unemployed, able to work	4 (2.1%)	7 (3.7%)	11 (2.9%)		
Unemployed, unable to work	1 (0.5%)	0 (0)	1 (0.3%)		
Manual labourer	7 (3.6%)^a^	18 (9.5%)^b^	25 (6.5%)		
**BMI**	25,7 (17.21-42.3)	27.39 (17.21-41.49)	26.33 (17.21: 42.3)	−3.363	**<0.001** ^**z**^
**Household size**	3 (23456789–10); mean rank = 179.84	3 (23456–7); mean rank = 205.42	3 (2: 10)	−2.258	**0.024** ^**z**^
**Presence of chronic disease**	40 (20.6%)	51 (26.8%)	91 (23.7%)	2.056	0.152^x^
**Current medication use**	33 (17%)	48 (25.3%)	81 (21.1%)	3.928	**0.048** ^**x**^
**Smoking status (None)**	112 (57.7%)	107 (56.3%)	219 (57%)	0.082	0.960^x^
**Alcohol use**					
No	112 (57.7%)^a^	132 (69.5%)^b^	244 (63.5%)	6.494	**0.043** ^**y**^
Regular	11 (5.7%)	11 (5.8%)	22 (5.7%)		
Occasionally	71 (36.6%)^a^	47 (24.7%)^b^	118 (30.7%)		
**Exercise status**					
No	71 (36.6%)	63 (33.2%)	134 (34.9%)	12.741	**0.004** ^**y**^
Regular	12 (6.2%)^a^	34 (17.9%)^b^	46 (12%)		
Occasionally	111 (57.2%)	93 (48.9%)	204 (53.1%)		

^x^Pearson’s chi-square test; ^y^Fisher’s exact test with Monte Carlo correction; ^z^Mann–Whitney U test; data are n (%) and median (min–max); mean rank; different superscript letters within the same row indicate statistically significant differences between air fryer ownership groups after post hoc comparison.

### Household division of food-related tasks by air-fryer ownership

Several food-related household tasks differed by air-fryer ownership (Table 2). For meal preparation, equal sharing with the spouse was more frequently reported among owners than non-owners (27.8% vs 17.4%; overall p = 0.042), and “other women in the household” were less frequently reported among owners (5.1% vs 11.1%). For table setting, equal sharing was markedly more common among owners (41.8% vs 23.7%) and “only self” and “other women in the household” were less common among owners (each 5.1% vs 13.7% and 13.2%, respectively; overall p < 0.001). For dishwashing, equal sharing was again more frequent among owners (29.4% vs 19.5%), while “other women in the household” was less frequent among owners (5.7% vs 14.7%; overall p = 0.010).

**Table 2:** Division of household food-related tasks by air fryer ownership (n, %).

	Air fryer Ownership Status		Total (%)	Test statistics	p-value
	Yes (%)	No (%)			
**Responsibility for meal preparation (cooking)**					
Self only	24 (12.4)	35 (18.4)	59 (15.4)	13.498	**0.042** ^**y**^
Mostly self	50 (25.8)	50 (26.3)	100 (26)		
Shared equally with spouse/partner	54 (27.8)^a^	33 (17.4)^b^	87 (22.7)		
Mostly spouse/partner	45 (23.2)	37 (19.5)	82 (21.3)		
Spouse/partner only	9 (4.6)	12 (6.3)	21 (5.5)		
Other women in the household	10 (5.1)^a^	21 (11.1)^b^	31 (8.1)		
Other men in the household	0 (0)	0 (0)	0 (0)		
Paid worker	0 (0)	1 (0.5)	1 (0.3)		
Other	2 (1)	1 (0.5)	3 (0.8)		
**Responsibility for table setting and clearing**					
Self only	10 (5.1)^a^	26 (13.7)^b^	36 (9.4)	27.515	**<0.001** ^**y**^
Mostly self	53 (27.3)	45 (23.7)	98 (25.5)		
Shared equally with spouse/partner	81 (41.8)^a^	45 (23.7)^b^	126 (32.8)		
Mostly spouse/partner	32 (16.5)	37 (19.5)	69 (18)		
Spouse/partner only	6 (3.1)	6 (3.2)	12 (3.1)		
Other women in the household	10 (5.1)^a^	25 (13.2)^b^	35 (9.1)		
Other men in the household	0 (0)	1 (0.5)	1 (0.3)		
Paid worker	0 (0)	1 (0.5)	1 (0.3)		
Other	2 (1)	4 (2.1)	6 (1.6)		
**Responsibility for dishwashing / loading the dishwasher**					
Self only	23 (11.9)	36 (19)	59 (15.4)	18.857	**0.010** ^**y**^
Mostly self	45 (23.2)	35 (18.4)	80 (20.8)		
Shared equally with spouse/partner	57 (29.4)^a^	37 (19.5)^b^	94 (24.5)		
Mostly spouse/partner	43 (22.2)	44 (23.2)	87 (22.7)		
Spouse/partner only	12 (6.2)	8 (4.2)	20 (5.2)		
Other women in the household	11 (5.7)^a^	28 (14.7)^b^	39 (10.2)		
Other men in the household	1 (0.5)	0 (0)	1 (0.3)		
Paid worker	0 (0)	1 (0.5)	1 (0.3)		
Other	2 (1)	1 (0.5)	3 (0.8)		

^x^Pearson’s chi-square test; ^y^Fisher’s exact test with Monte Carlo correction; ^z^Mann–Whitney U test; data are n (%) and median (min–max)/mean rank; same letters indicate no significant difference.

### Sex-stratified differences in culinary skills and Mediterranean diet adherence within each air-fryer group

Within both ownership strata, women had higher scores than men across most cooking and food preparation skill domains (all p < 0.001), including cooking method, food preparation techniques, total cooking skills, meal planning/preparation, shopping, “skillfulness,” label reading/consumer awareness, and total food preparation skills (Table 3). Budgeting did not differ by sex among owners (p = 0.344) or non-owners (p = 0.884).

**Table 3:** Sex-stratified comparison of cooking skills, food preparation skills, and Mediterranean diet adherence scores among air fryer owners and non-owners.

	Air fryer Ownership (+)				Air fryer Ownership (-)			
	Sex		Test statistic	p-value	Sex		Test statistic	p-value
	Male	Female			Male	Female		
**COOKING AND FOOD PREPARATION SKILLS SCALE**								
**Cooking Skills**								
Cooking methods subscale score	38.5 (0-56)	50.5 (0-56)	−6.546	**<0.001** ^**x**^	32 (0-56)	49 (11-56)	−7.003	**<0.001** ^**x**^
Food preparation techniques subscale score	24 (0-42)	33 (14-42)	−6.398	**<0.001** ^**x**^	19 (0-42)	36 (12-42)	−8.383	**<0.001** ^**x**^
Total cooking skills score	60.5 (0-98)	84.5 (14-98)	−7.151	**<0.001** ^**x**^	52 (0-98)	84 (39-98)	−8.089	**<0.001** ^**x**^
**Food Preparation Skills**								
Meal planning and preparation subscale score	9 (0-21)	16 (0-21)	−5.503	**<0.001** ^**x**^	7.5 (0-21)	15 (0-21)	−5.789	**<0.001** ^**x**^
Shopping subscale score	14 (0-21)	18 (0-21)	−3.261	**0.001** ^**x**^	15 (0-21)	18 (0-21)	−3.553	**<0.001** ^**x**^
Budgeting subscale score	24 (0-28)	21.5 (6-28)	−0.946	0.344^x^	24 (0-28)	24 (0-28)	−0.146	0.884^x^
Resourcefulness subscale score	19.5 (0-35)	33 (0-35)	−6.99	**<0.001** ^**x**^	16.5 (0-35)	30 (0-35)	−7.783	**<0.001** ^**x**^
Food label reading / consumer awareness subscale score	19.5 (0-28)	22 (0-28)	−2.648	**0.008** ^**x**^	18.5 (0-28)	23 (0-28)	−2.401	**0.016** ^**x**^
Total food preparation skills score	83 (0-133)	105.5 (35-133)	−6.42	**<0.001** ^**x**^	75 (0-133)	106 (57-133)	−6.951	**<0.001** ^**x**^
**MEDITERRANEAN DIET ADHERENCE SCREENER** **(MEDAS)**								
MEDAS Total Score	5 (234567891011–12)	8 (2345678910–11)	−5.868	**<0.001** ^**x**^	6 (12345678910–11)	7 (1234567891011–12)	−2.716	**0.007** ^**x**^
MEDAS Categories								
No adherence	71 (74%)^a^	31 (31.6%)^b^	35.402	**<0.001** ^**y**^	56 (58.3%)	41 (43.6%)	4.352	0.112^y^
Acceptable adherence (7 ≤ MEDAS <9)	14 (14.6%)^a^	38 (38.8%)^b^			27 (28.1%)	33 (35.1%)		
High/strict adherence (MEDAS ≥9)	11 (11.5%)^a^	29 (29.6%)^b^			13 (13.5%)	20 (21.3%)		

Data for scale scores are presented as median (minimum–maximum). MEDAS categories are presented as n (%). ^x^Mann–Whitney U test; ^y^Fisher’s exact test with Monte Carlo correction. Different superscript letters within the same row indicate statistically significant differences between male and female participants within the same air fryer ownership group. MEDAS, Mediterranean Diet Adherence Screener.

Mediterranean diet adherence (MEDAS) differed by sex in both strata. Among owners, women had higher MEDAS scores than men (median 8 [2–11] vs 5 [2–12]; p < 0.001) and the categorical MEDAS distribution differed significantly by sex (p < 0.001): “no adherence” was more common among men (74.0%) than women (31.6%), while acceptable/strict adherence were also more common among women (38.8% and 29.6%, respectively) than men (14.6% and 11.5%). Among non-owners, women also had higher MEDAS scores than men (median 7 [1–12] vs 6 [1–11]; p = 0.007), but the categorical distribution did not differ significantly by sex (p = 0.112).

By air fryer ownership status, overall cooking skills did not differ between owners and non-owners, including cooking methods (median 44 [0–56] vs 42.5 [0–56]; p = 0.114), food preparation techniques (30 [0–42] vs 28 [0–42]; p = 0.529), and total cooking skills score (74 [0–98] vs 73 [0–98]; p = 0.260). Similarly, MEDAS scores (median 6 [2–12] vs 6 [1–12]; p = 0.664) and MEDAS categories did not differ by ownership (p = 0.515). Among food preparation skills, most subdomains and the total food preparation skills score were comparable between groups (total score: 94.5 [0–133] vs 90.5 [0–133]; p = 0.150), although resourcefulness was slightly higher among owners (28 [0–35] vs 26 [0–35]; p = 0.047).

### Factors associated with Mediterranean diet adherence (MEDAS score)

**Fig 2:**
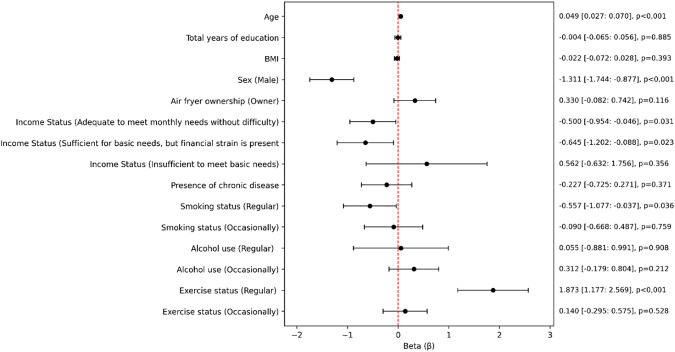
Robust regression coefficient plot for factors associated with Mediterranean diet adherence score. Points represent unstandardized regression coefficients, and horizontal lines represent 95% confidence intervals. The red dashed vertical line indicates β = 0.

In Table 4, the robust regression model for MEDAS was statistically significant (F = 8.802; p < 0.001; R² = 26.40%; Durbin–Watson = 1.866). Higher age was associated with higher MEDAS score (β = 0.049; 95% CI: 0.027 to 0.070; p < 0.001). Male sex was associated with a lower MEDAS score (β=−1.311; 95% CI: −1.744 to −0.877; p < 0.001). Compared with the reference income category (“More than adequate to comfortably cover monthly expenses”), two income categories had lower MEDAS: “Adequate to meet monthly needs without difficulty” (β=−0.500; 95% CI: −0.954 to −0.046; p = 0.031) and “Sufficient for basic needs, but financial strain is present” (β=−0.645; 95% CI: −1.202 to −0.088; p = 0.023). Regular smoking (vs none) was associated with lower MEDAS (β=−0.557; 95% CI: −1.077 to −0.037; p = 0.036), while regular exercise (vs none) was associated with higher MEDAS (β = 1.873; 95% CI: 1.177 to 2.569; p < 0.001). Air-fryer ownership was not significantly associated with MEDAS in this model (β = 0.330; 95% CI: −0.082 to 0.742; p = 0.116). The direction and magnitude of the regression coefficients for factors associated with Mediterranean diet adherence are visually summarized in Fig 2.

**Table 4:** Factors associated with Mediterranean diet adherence (MEDAS score): robust regression results.

	β^1^ (95% CI)	S. Error	β^2^	t	p-value	VIF
Constant	5.95 (4.006: 7.894)	0.988	0.000	6.019	**<0.001**	
Age	0.049 (0.027: 0.07)	0.011	0.247	4.406	**<0.001**	1.568
Total years of education	−0.004 (−0.065: 0.056)	0.031	−0.009	−0.145	0.885	1.737
BMI	−0.022 (−0.072: 0.028)	0.026	−0.044	−0.855	0.393	1.323
Sex (Male)	−1.311 (−1.744: −0.877)	0.221	−0.298	−5.944	**<0.001**	1.255
Air fryer ownership (Owner)	0.33 (−0.082: 0.742)	0.210	0.075	1.574	0.116	1.134
Income Status (Adequate to meet monthly needs without difficulty)	−0.5 (−0.954: −0.046)	0.231	−0.113	−2.164	**0.031**	1.360
Income Status (Sufficient for basic needs, but financial strain is present	−0.645 (−1.202: −0.088)	0.283	−0.118	−2.277	**0.023**	1.331
Income Status (Insufficient to meet basic needs)	0.562 (−0.632: 1.756)	0.607	0.044	0.925	0.356	1.111
Presence of chronic disease	−0.227 (−0.725: 0.271)	0.253	−0.044	−0.895	0.371	1.204
Smoking status (Regular)	−0.557 (−1.077: −0.037)	0.264	−0.114	−2.108	**0.036**	1.453
Smoking status (Occasionally)	−0.09 (−0.668: 0.487)	0.294	−0.015	−0.307	0.759	1.127
Alcohol use (Regular)	0.055 (−0.881: 0.991)	0.476	0.006	0.116	0.908	1.262
Alcohol use (Occasionally)	0.312 (−0.179: 0.804)	0.250	0.065	1.250	0.212	1.367
Exercise status (Regular)	1.873 (1.177: 2.569)	0.354	0.275	5.291	**<0.001**	1.353
Exercise status (Occasionally)	0.14 (−0.295: 0.575)	0.221	0.032	0.632	0.528	1.259

F = 8.802; p < 0.001; R^2^ = 26.40%; Durbin-Watson = 1.866; ¹Unstandardized beta coefficient; ²Standardized beta coefficient; VIF: Variance Inflation Factor.

### Factors associated with cooking and food preparation skills

Cooking-related competencies were assessed using the Cooking and Food Preparation Skills Scale, and determinants of the two subscale outcomes were examined using robust regression models. For the Cooking Skills subscale, the model was statistically significant (F = 17.403; p < 0.001; R² = 41.50%; Durbin–Watson = 1.722) (Table 5). Older age was associated with higher cooking skills (β = 0.504; 95% CI: 0.304 to 0.704; p < 0.001), whereas male sex was associated with substantially lower cooking skills (β=−26.400; 95% CI: −30.375 to −22.425; p < 0.001). Air-fryer ownership was positively associated with cooking skills (β = 4.253; 95% CI: 0.469 to 8.036; p = 0.028), while presence of chronic disease was associated with lower cooking skills (β=−4.824; 95% CI: −9.326 to −0.322; p = 0.036).

**Table 5:** Factors associated with total cooking skills score: robust regression results.

	β^1^ (95% CI)	S. Error	β^2^	t	p-value	VIF
Constant	61.414 (43.742: 79.086)	8.987	0.000	6.834	**<0.001**	
Age	0.504 (0.304: 0.704)	0.102	0.249	4.950	**<0.001**	1.591
Total years of education	0.356 (−0.189: 0.901)	0.277	0.068	1.285	0.200	1.751
BMI	−0.137 (−0.598: 0.323)	0.234	−0.027	−0.586	0.558	1.350
Sex (Male)	−26.4 (−30.375: −22.425)	2.022	−0.586	−13.059	**<0.001**	1.268
Air fryer ownership (Owner)	4.253 (0.469: 8.036)	1.924	0.095	2.210	**0.028**	1.151
Income Status (Adequate to meet monthly needs without difficulty)	−2.098 (−6.227: 2.031)	2.100	−0.046	−0.999	0.318	1.356
Income Status (Sufficient for basic needs, but financial strain is present	−1.236 (−6.32: 3.848)	2.585	−0.022	−0.478	0.633	1.322
Income Status (Insufficient to meet basic needs)	−9.901 (−21.313: 1.511)	5.803	−0.072	−1.706	0.089	1.106
Presence of chronic disease	−4.824 (−9.326: −0.322)	2.289	−0.092	−2.107	**0.036**	1.203
Smoking status (Regular)	−2.64 (−7.382: 2.102)	2.411	−0.052	−1.095	0.274	1.434
Smoking status (Occasionally)	0.317 (−4.945: 5.578)	2.676	0.005	0.118	0.906	1.138
Alcohol use (Regular)	1.984 (−6.938: 10.906)	4.537	0.020	0.437	0.662	1.260
Alcohol use (Occasionally)	−0.153 (−4.611: 4.304)	2.267	−0.003	−0.068	0.946	1.360
Exercise status (Regular)	3.26 (−3.111: 9.632)	3.240	0.047	1.006	0.315	1.386
Exercise status (Occasionally)	3.646 (−0.362: 7.655)	2.038	0.081	1.789	0.075	1.285

F = 17.403; p < 0.001; R^2^ = 41.50%; Durbin-Watson = 1.722; ¹Unstandardized beta coefficient; ²Standardized beta coefficient; VIF: Variance Inflation Factor.

**Fig 3:**
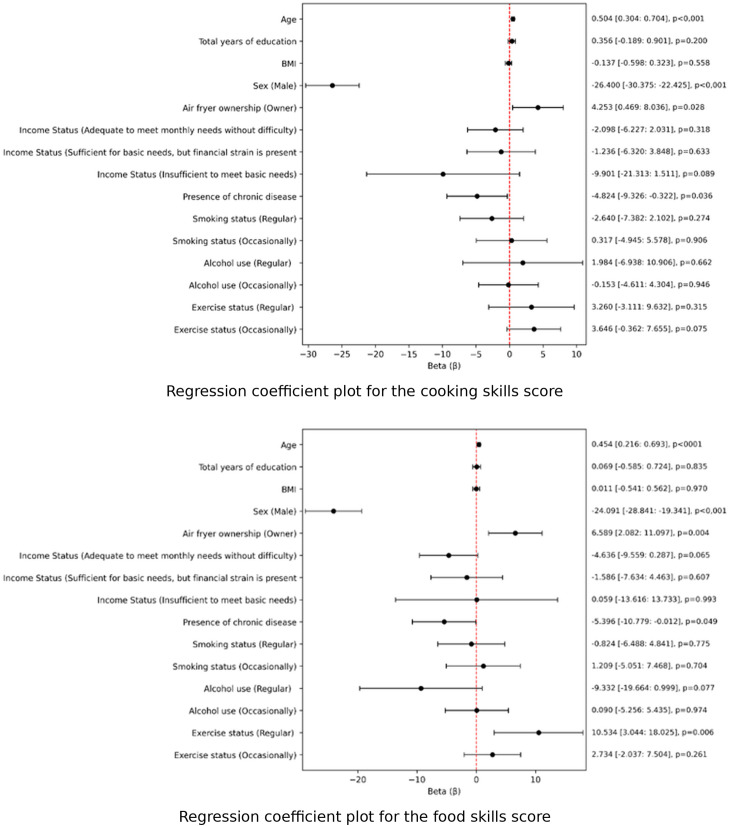
Robust regression coefficient plots for cooking skills and food preparation skills scores. Points represent unstandardized regression coefficients, and horizontal lines represent 95% confidence intervals. The red dashed vertical line indicates β = 0. Values to the right of zero indicate positive associations, whereas values to the left indicate negative associations. Panel A shows the robust regression model for the cooking skills score, and Panel B shows the robust regression model for the food preparation skills score.

For the Food Preparation Skills subscale, the model was also statistically significant (F = 11.549; p < 0.001; R² = 32.01%; Durbin–Watson = 1.621) (Table 6). Older age was associated with higher food preparation skills (β = 0.454; 95% CI: 0.216 to 0.693; p < 0.001), while male sex was associated with lower food preparation skills (β=−24.091; 95% CI: −28.841 to −19.341; p < 0.001). Air-fryer ownership remained positively associated with food preparation skills (β = 6.589; 95% CI: 2.082 to 11.097; p = 0.004). Presence of chronic disease was associated with lower food preparation skills (β=−5.396; 95% CI: −10.779 to −0.012; p = 0.049), and regular exercise (vs none) was associated with higher food preparation skills (β = 10.534; 95% CI: 3.044 to 18.025; p = 0.006). Fig 3 provides a visual summary of the robust regression coefficients and 95% confidence intervals for the factors associated with cooking skills and food preparation skills scores.

**Table 6:** Factors associated with food preparation skills score: robust regression results.

	β^1^ (95% CI)	S. Error	β^2^	t	p-value	VIF
Constant	83.002 (61.88: 104.125)	10.742	0.000	7.727	**<0.001**	
Age	0.454 (0.216: 0.693)	0.121	0.202	3.747	**<0.001**	1.579
Total years of education	0.069 (−0.585: 0.724)	0.333	0.012	0.208	0.835	1.760
BMI	0.011 (−0.541: 0.562)	0.280	0.002	0.037	0.970	1.353
Sex (Male)	−24.091 (−28.841: −19.341)	2.415	−0.484	−9.974	**<0.001**	1.273
Air fryer ownership (Owner)	6.589 (2.082: 11.097)	2.292	0.132	2.875	**0.004**	1.148
Income Status (Adequate to meet monthly needs without difficulty)	−4.636 (−9.559: 0.287)	2.504	−0.093	−1.852	0.065	1.353
Income Status (Sufficient for basic needs, but financial strain is present	−1.586 (−7.634: 4.463)	3.076	−0.026	−0.515	0.607	1.324
Income Status (Insufficient to meet basic needs)	0.059 (−13.616: 13.733)	6.954	0.000	0.008	0.993	1.101
Presence of chronic disease	−5.396 (−10.779: −0.012)	2.738	−0.093	−1.971	**0.049**	1.196
Smoking status (Regular)	−0.824 (−6.488: 4.841)	2.881	−0.015	−0.286	0.775	1.431
Smoking status (Occasionally)	1.209 (−5.051: 7.468)	3.183	0.017	0.380	0.704	1.132
Alcohol use (Regular)	−9.332 (−19.664: 0.999)	5.254	−0.086	−1.776	0.077	1.263
Alcohol use (Occasionally)	0.09 (−5.256: 5.435)	2.718	0.002	0.033	0.974	1.367
Exercise status (Regular)	10.534 (3.044: 18.025)	3.809	0.139	2.765	**0.006**	1.371
Exercise status (Occasionally)	2.734 (−2.037: 7.504)	2.426	0.055	1.127	0.261	1.280

F = 11.549; p < 0.001; R^2^ = 32.01%; Durbin-Watson = 1.621; ¹Unstandardized beta coefficient; ²Standardized beta coefficient; VIF: Variance Inflation Factor.

## Discussion

In this cross-sectional sample of cohabiting adults in Istanbul, air fryer ownership was associated with a household context in which equal sharing was reported more often for some routine kitchen tasks, although pronounced sex differences persisted in cooking and food preparation skills and Mediterranean diet adherence. After adjustment for sociodemographic and health behavior factors, robust regression indicated that ownership was associated with modestly higher cooking and food preparation skill scores, whereas no association was observed with MEDAS. Mediterranean diet adherence remained strongly socially patterned, being higher with older age and regular exercise and lower among men, those reporting lower income, and regular smokers, reinforcing that overall diet quality is shaped primarily by social position and health practices rather than by kitchen technology itself.

Air fryer ownership likely acted as a marker of household culinary responsibility organisation and technology adoption rather than a direct dietary exposure. The positive association between ownership and higher skills scores in adjusted models could reflect non-causal explanations, including perceived lower task complexity that may coincide with greater participation in cooking, as well as clustering with unmeasured attributes such as interest in kitchen devices, time scarcity, socioeconomic position, or preference for quick home meal preparation. Because cooking and food skills scales capture perceived capability across techniques, planning, shopping, resourcefulness, and label reading, greater day-to-day engagement with food preparation may be linked to higher perceived competence even when the appliance itself standardizes parts of the cooking process [5].

The absence of an association between ownership and Mediterranean diet adherence was also epidemiologically plausible, since MEDAS reflects habitual intake patterns rather than competence alone [16]. Skills may be associated with healthier eating, but dietary choices remain shaped by costs, time constraints, preferences, and food availability, and convenience technologies can support preparation of both minimally processed foods and ultra-processed ready to cook products [22]. Prior evidence indicates that changes in skills do not always yield meaningful improvements in diet quality, and that ultra-processed diets can increase energy intake and weight gain even when nutrient profiles are matched, underscoring that food form and processing matter beyond cooking method [23,24]. The strong inverse associations of male sex with both skills outcomes and MEDAS further suggested that gendered roles and socialization remained central determinants of household food practices, and that greater task sharing does not necessarily imply equal competence or equal cognitive responsibility for food-related work. Similarly, the widespread availability of online recipes and cooking information may reduce informational barriers to cooking, but it does not necessarily alter who assumes responsibility for planning, preparing, and coordinating meals within the household.

The present findings align with evidence linking more frequent home cooking and stronger cooking and food preparation skills with healthier dietary patterns, while also indicating that these relationships depend on what foods are prepared and under what socioeconomic constraints [3,23,25–27]. Greater cooking frequency has been associated with better diet quality and, in some settings, lower adiposity, although these benefits may vary by available resources, time, and food access [3,25–27]. Likewise, higher cooking and food preparation skills are often associated with healthier dietary patterns, but the evidence is limited by cross-sectional designs, heterogeneous measures, and intervention findings showing that skill gains do not always translate into sustained dietary improvement [23,28]. This is consistent with the present results, in which air fryer ownership was associated with higher skills scores but not with Mediterranean diet adherence.

The observed tendency toward more equal sharing of selected kitchen tasks among owners also fit with sociological work on domestic technology and unpaid labour. Time use research indicates that labour saving household technologies do not necessarily reduce women’s total unpaid work proportionally and may instead reshape how tasks are sequenced and managed without eliminating underlying responsibility [10]. Historical econometric evidence has further suggested that appliance diffusion can alter time allocation in specific contexts, yet changes in gendered divisions of unpaid work have generally been gradual and incomplete [11]. Against this background, the present results suggest that a contemporary convenience appliance may coincide with greater task sharing in some visible domains, while persistent sex differences in skills and diet adherence point to enduring gendered roles and the uneven distribution of responsibility for culinary responsibility.

Determinants of MEDAS were also consistent with established epidemiology of healthier dietary patterns. Higher adherence with older age and regular exercise, and lower adherence among regular smokers, followed common clustering of health behaviours [29–31]. The association between lower perceived income and lower adherence further supported the broader evidence that socioeconomic disadvantage constrains access to healthier foods and is linked to less favorable dietary patterns [32,33].

The magnitude of sex differences in cooking and food preparation skills observed in this study was coherent with national and international time use evidence showing that unpaid domestic and care labour remains strongly gendered. In Turkish time use statistics, women spend substantially more time than men on household and family care, and analyses of “time poverty” indicate that women’s unpaid work burden remains higher across socioeconomic strata, even when absolute unpaid time decreases with higher education or income [34,35]. This macro level context offers a plausible explanation for why men in both ownership strata had markedly lower cooking and food preparation skills and lower Mediterranean diet adherence. It also supports the interpretation that the more frequent equal sharing of selected tasks reported by air fryer owners should not be attributed to the technology itself; rather, ownership likely marked a socially patterned subgroup with greater resources and potentially different household norms that may coincide with some redistribution of visible tasks. However, evidence from gender scholarship emphasizes that domestic inequality extends beyond task execution to cognitive labour such as planning, coordinating, and monitoring, which often remains feminized even when task sharing appears more balanced [36].

These findings are consistent with the view that cooking and food preparation skills are core life and health skills and that intervention priorities should center on capabilities and household organization rather than on promoting specific devices. Skill development is likely most effective when it begins early and is developmentally appropriate, using age aligned task frameworks to guide curricula and caregiver support [37], and when it covers both cooking techniques and broader food skills such as meal planning, shopping, budgeting, resourcefulness, and label reading [5,23]. Adult cooking programs can improve skills and selected psychosocial and dietary outcomes, although previous research studies have emphasized the need for stronger designs and approaches that support maintenance over time [28,38]. A gender equity lens is essential, because interventions that implicitly assume a single household cook risk reinforcing women’s unpaid work and mental load; the observed sex gaps in skills and adherence suggest that future strategies could address men’s competence and responsibility while also addressing the cognitive labour of planning and coordination as part of equitable culinary accountability [36]. These findings should not be interpreted as evidence that air frying is inherently health promoting, because nutritional effects depend on the foods prepared and cooking practices used. Guidance should continue to emphasize minimally processed foods and avoidance of excessive browning regardless of appliance type [39–41].

Strengths include a cohabiting-household design with one respondent per household and validated instruments for cooking/food skills and Mediterranean diet adherence [5,16,42]. The main added value is the integration of air fryer ownership, gendered kitchen labour division, and Mediterranean diet adherence in a single framework. Robust regression offered resilience to non-normality and outliers while allowing adjustment for multiple sociodemographic and health behavior covariates. Important limitations remained. First, the cross-sectional design precluded causal inference and could not establish whether air fryer ownership preceded differences in task sharing, skills, or diet. Reverse causality also cannot be excluded; for example, individuals or households with greater interest in cooking, higher food preparation skills, or more egalitarian kitchen routines may have been more likely to acquire an air fryer. Second, the use of snowball sampling in a single metropolitan setting limited representativeness and may have introduced selection bias. Participants reached through this recruitment approach may differ from the broader population of cohabiting adults in Istanbul or Türkiye in socioeconomic position, interest in kitchen technologies, health behaviours, and household food-related norms. Therefore, the findings should not be generalised beyond the sampled population. All measures were self-reported, including task allocation and perceived skills, raising the possibility of social desirability bias, and household practices were reported by only one partner, which could have introduced systematic measurement error if partner perceptions differed. Residual confounding was also likely, including unmeasured frequency and purpose of air fryer use, foods prepared, time scarcity, nutrition knowledge, and purchasing of ultra-processed foods, and MEDAS is a brief screener that does not capture dietary complexity such as portion sizes, cooking frequency, or detailed ultra-processed food intake. Future research would benefit from longitudinal and dyadic designs incorporating both partners, alongside mixed methods work to examine household bargaining, norms, and cognitive labour in meal planning and how appliances are integrated into everyday domestic culinary labour; more granular dietary assessment could test whether air fryer use displaces deep fat frying, increases reliance on ready to cook products, or supports preparation of vegetables and legumes, including associations with UPF intake and objective nutritional markers, and interventions that build cooking and food skills while explicitly targeting equitable task sharing could evaluate whether skills gains translate into improved diet quality and reduced mental load for women.

## Conclusions

In this cross-sectional sample of cohabiting adults in Istanbul, air fryer ownership aligned with more equal sharing of several routine kitchen tasks and was independently associated with modestly higher cooking and food preparation skills, yet it was not associated with greater Mediterranean diet adherence. In this sample, Mediterranean diet adherence was socially patterned by age, sex, perceived income, smoking, and exercise, suggesting that diet quality may be shaped more by social position and health practices than by cooking technology itself. These findings suggest the relevance of a policy and practice focus on early and equitable development of cooking and food skills, alongside household-level approaches that address both the execution and the cognitive labour of culinary responsibility, rather than promoting specific kitchen technologies as nutrition interventions.

## Supporting information

S1 File Airfryer. (CSV)
