# Supplementary material for: Air fryer ownership, household food-related task sharing, cooking skills, and mediterranean diet adherence among cohabiting adults in Türkiye: A cross-sectional study
Source: PLoS One. 2026 Jul 8;21(7):e0352090. doi: 10.1371/journal.pone.0352090 (PMC13345407; doi:10.1371/journal.pone.0352090)
Supplement: Attachment — Submitted filename: Reviewer Confidential Comments to Editor.pdf [file pone.0352090.s002.pdf]

Confidential Comments to Editor

1. Age group of study participants?
2. Well, study seems pretty interesting and interactive. I think authors might have lacked the proper layout/study design of this behavioral study.
3. To my surprise there is no figure in the whole research article.
4. Comparative Figures may be more impactful and easy to understand along with a few tables.
5. Tables contain more detailed statistical data parameters, which I think are no more required in modern day article analysis. Yes, you do need a significance statement or decision making.
6. What authors are trying to evaluate from this study? Air fryer ownership effect on the behavior of users and their diet pattern? Well, if you are focusing on air fryer, then you should specifically get data for affordability of getting it? If users are unable to buy one, what are the factors behind it? You are studying a specific location, please specify that circumference, it should not be like study in Study. Is 384 nos. is the true representative of the subject population? What is meant by the air fryer ownership? Did authors check that to which individual the air fryer was registered? OR who purchased it? If both partners shared it then what is the criteria?
7. Moreover, it is evident that women are better cook then men and they have better esthetic sense than men too.
8. Education should be mentioned numerically like masters or bachelors or primary education etc.
9. Today is the age of technology, every recipe is available on net. So, anyone can check and follow it from there to cook anything. Many more questions are discussable.

Reviewer's Judgement:

I am afraid about the feasibility or suitability of the paper for your journal and I don't **recommend** it for publication please.

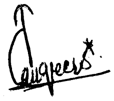

Tauqeer Ahmad
Senior Scientist
PAEC, Pakistan
