# Supplementary material for: Air fryer ownership, household food-related task sharing, cooking skills, and mediterranean diet adherence among cohabiting adults in Türkiye: A cross-sectional study
Source: PLoS One. 2026 Jul 8;21(7):e0352090. doi: 10.1371/journal.pone.0352090 (PMC13345407; doi:10.1371/journal.pone.0352090)
Supplement: Attachment — Submitted filename: Response_to_Reviewers_airfryer_zbka.docx [file pone.0352090.s003.docx]

**Submission ID:** PONE-D-26-12396

**Title:** Air fryer ownership, household food-related task sharing, cooking skills, and mediterranean diet adherence among cohabiting adults in Türkiye: a cross-sectional study

**Journal:** PLOS One

**Response:** As authors, we would like to extend our sincerest gratitude to the editor and the reviewers for their valuable recommendations. Please see below an itemized table of each of reviewers’ comments and answers for each comment separately.

| **Editor’s** **Comments and Suggestions for Author** | **Author’s response** |
| --- | --- |
| Thank you for submitting your manuscript to PLOS ONE. After careful consideration, we feel that it has merit but does not fully meet PLOS ONE’s publication criteria as it currently stands. Therefore, we invite you to submit a revised version of the manuscript that addresses the points raised during the review process. | We sincerely thank the Academic Editor for the careful evaluation of our manuscript and for the opportunity to submit a revised version. We are grateful that the manuscript was considered to have merit, and we greatly appreciate the constructive guidance provided to improve its clarity, rigor, and interpretability. We have carefully revised the manuscript in response to the Editor’s comments and the reviewers’ suggestions, with particular attention to improving the presentation of Table 3, clarifying the conceptual framing, strengthening the discussion of sampling and generalizability, avoiding causal language, and enhancing the visual presentation of the study. In addition, we have reformatted the manuscript according to the PLOS ONE templates and style requirements provided in the decision letter. We hope that the revised version now addresses the concerns raised and better communicates the contribution of the study to the literature on household food practices, cooking technologies, and diet-related behaviors. Below, we provide a detailed point-by-point response to each comment. |
| This manuscript addresses an interesting and underexplored topic by integrating household technology use with food-related behaviors and dietary outcomes. The study is well motivated, and the use of validated tools alongside regression modelling strengthens the analytical approach. However, several important issues should be addressed to improve clarity, rigor, and interpretability. | We thank the Academic Editor for this encouraging and constructive assessment. In response, we have revised the manuscript with the aim of improving clarity, methodological transparency, and interpretability. The main revisions include clearer conceptual framing, improved presentation of the results, stricter non-causal wording, expanded discussion of sampling limitations, and the addition of visual material to support readability. |
| **Major Issues** | **Author’s response** |
| Clarity and Consistency of Data Presentation (Table 3)  Table 3 is currently difficult to interpret and may be misleading. The reported values appear inconsistent with the stated sample size (N=384), which creates confusion regarding whether the values represent counts, medians, or scale scores. This issue was also identified by another reviewer and must be addressed.  Clearly specify the unit of analysis (e.g., median [min–max], score, or n).  Ensure that totals are not misinterpreted as participant counts.  Consider restructuring the table for readability (e.g., separating subscales or presenting summary scores only). | We thank the Academic Editor for this important comment. We agree that the previous presentation of Table 3 could have allowed scale scores to be misinterpreted as participant counts. We therefore revised the table title, row labels, and footnote to explicitly state that cooking skills, food preparation skills, and MEDAS total values are scale scores presented as median (minimum–maximum), whereas MEDAS categories are presented as n (%). We also added “score” to the relevant row labels and separated “MEDAS Total Score” from “MEDAS Categories” to improve readability and prevent misinterpretation. The values were carefully rechecked, and no calculation error was identified. |
| Interpretation of Associations (Causality)  The manuscript occasionally implies directional or causal relationships (e.g., ownership influencing skills or behaviors). Given the cross-sectional design, all findings should be framed strictly as associations.  Revise wording throughout (especially Discussion) to avoid causal language.  Emphasize potential reverse causality and residual confounding. | We thank the Academic Editor for this important comment. We agree that, given the cross-sectional design, all findings should be framed strictly as associations rather than causal or directional relationships. We therefore reviewed the manuscript throughout, with particular attention to the Introduction, Discussion, and Conclusions, and revised wording that could imply causality. Terms suggesting directionality or mechanism, such as “enabled,” “facilitate,” and “determinants,” were replaced with more cautious language such as “was associated with,” “may coincide with,” “may reflect,” and “factors associated with.”  We also clarified the interpretation of air fryer ownership as a possible marker of household technology adoption, socioeconomic positioning, and domestic food-practice patterns rather than as a causal exposure. The limitations section also explicitly notes that causal inference cannot be made, temporality cannot be established, reverse causality cannot be excluded, and residual confounding is possible. |
| Sampling Strategy and Generalizability  The use of snowball sampling limits representativeness and introduces selection bias.  This limitation should be more explicitly acknowledged.  Avoid generalizing findings beyond the sampled population. | We thank the Academic Editor for this important comment. We agree that the use of snowball sampling limits representativeness and may have introduced selection bias. In response, we revised the manuscript to acknowledge this limitation more explicitly and to avoid generalizing the findings beyond the sampled population. Specifically, we clarified that the sample should not be considered statistically representative of all cohabiting adults in Istanbul or Türkiye. We also expanded the limitations section to note that participants reached through snowball sampling may differ from the broader population in socioeconomic position, interest in kitchen technologies, health behaviors, and household food-related norms. We also amended the Conclusion section to further restrict the interpretation of the findings to the sampled population and to avoid broader generalizations. Accordingly, the findings are now framed as applying to the surveyed cohabiting adults in Istanbul rather than to the wider population. |
| Conceptual Alignment of Study Objectives  The manuscript combines multiple constructs (technology ownership, task sharing, skills, and diet adherence), but the conceptual linkage between them could be more explicitly articulated.  Clarify whether air fryer ownership is conceptualized as a proxy for lifestyle, convenience behavior, or socioeconomic positioning.  Strengthen the theoretical rationale linking these domains. | We thank the Academic Editor for this important comment. We agree that the conceptual linkage among technology ownership, household task sharing, cooking and food preparation skills, and Mediterranean diet adherence needed to be articulated more explicitly. In response, we revised the Introduction to clarify that air fryer ownership is conceptualized not as a direct nutritional exposure or inherently health-promoting technology, but as a household-level marker of contemporary kitchen technology adoption. We now explain that ownership may reflect overlapping dimensions of convenience-oriented food preparation, socioeconomic positioning and purchasing capacity, interest in cooking technologies and home food preparation, household organization of culinary labor, and broader lifestyle or health-behavior patterns. We also strengthened the rationale for examining task sharing, cooking/food preparation skills, and Mediterranean diet adherence together as interrelated components of domestic food practices. Finally, we revised the study aim to clarify that the purpose was to situate air fryer ownership within broader domestic food-practice patterns, rather than to evaluate the appliance as a causal determinant of diet quality, skills, or household labor division. |
| **Minor Issues** | **Author’s response** |
| Figures and Visual Presentation  Although not mandatory, the inclusion of at least one figure would improve clarity.  Suggested: conceptual framework or regression coefficient plot. | We thank the Academic Editor for this helpful suggestion. In response, we added three figures to improve the clarity and visual presentation of the manuscript: a study flow diagram summarizing the study design, eligibility criteria, final analytic sample, ownership groups, and survey measures; a regression coefficient plot for the Mediterranean diet adherence model; and paired regression coefficient plots for the cooking skills and food preparation skills models. These figures visually summarize key methodological and analytical components and complement the tabular presentation of the findings. |
| Statistical Reporting  Ensure consistent reporting format across tables (especially medians vs means).  Clarify all test statistics and notation in table footnotes. | We thank the Academic Editor for this important comment. We have carefully reviewed the tables to improve consistency in statistical reporting. In the revised manuscript, continuous non-normally distributed variables are consistently presented as median (minimum–maximum), while categorical variables are presented as n (%). We also revised the table footnotes to clarify the statistical tests used, including Pearson’s chi-square test, Fisher’s exact test with Monte Carlo correction, and the Mann–Whitney U test, and to explain the meaning of superscript letters where post hoc comparisons were performed. We will ensure that all tables are checked again before resubmission for consistency in decimal formatting, p-value presentation, test statistics, and notation. |
| Referencing Style  Some references use “et al.” inconsistently; ensure alignment with journal guidelines. | We thank the Academic Editor for this comment. We have reviewed the reference list and revised the formatting to ensure consistency with PLOS ONE reference style. In particular, we checked the use of “et al.” and will ensure that author names, journal titles, year, volume, issue, and page/article numbers are presented consistently throughout the reference list before resubmission. |
| Language and Style  Minor grammatical and stylistic edits are needed to improve clarity and readability. | We thank the Academic Editor for this comment. We carefully reviewed the manuscript for grammar, style, and readability and made minor language edits throughout. These revisions included removing repetitive wording, improving sentence flow, correcting typographical errors, and standardizing terminology across the Abstract, Introduction, Results, Discussion, tables, and footnotes. We also checked the revised text to ensure that the language remained clear, cautious, and appropriate for a cross-sectional study. |
| **Journal Requirements:** | **Author’s response** |
| 1. Please ensure that your manuscript meets PLOS ONE's style requirements, including those for file naming. The PLOS ONE style templates can be found at  https://journals.plos.org/plosone/s/file?id=wjVg/PLOSOne_formatting_sample_main_body.pdf and  https://journals.plos.org/plosone/s/file?id=ba62/PLOSOne_formatting_sample_title_authors_affiliations.pdf | We thank the journal office for this guidance. We have reformatted the manuscript according to the PLOS ONE style requirements and the templates provided in the decision letter. We have also checked the file names and will ensure that the clean revised manuscript, the highlighted revised manuscript, response to reviewers, figures, and any supporting files are uploaded using appropriate file names in line with PLOS ONE requirements. |
| 2. Please provide additional details regarding participant consent. In the ethics statement in the Methods and online submission information, please ensure that you have specified (1) whether consent was informed and (2) what type you obtained (for instance, written or verbal, and if verbal, how it was documented and witnessed). If your study included minors, state whether you obtained consent from parents or guardians. If the need for consent was waived by the ethics committee, please include this information.  If you are reporting a retrospective study of medical records or archived samples, please ensure that you have discussed whether all data were fully anonymized before you accessed them and/or whether the IRB or ethics committee waived the requirement for informed consent. If patients provided informed written consent to have data from their medical records used in research, please include this information. | We thank the journal office for this comment. We have revised the ethics statement in the Methods section and the online submission information to provide additional details regarding participant consent. Specifically, we clarified that all participants were adults and that written informed consent was obtained from each participant before data collection. No minors were included in the study.  We thank the journal office for this clarification. This study was not a retrospective study of medical records or archived samples. Data were collected directly from adult participants using a structured questionnaire after written informed consent was obtained. Therefore, no retrospective medical record data or archived samples were used. |
| 3. Thank you for stating the following financial disclosure:  “This research was partially funded by the Scientific and Technological Research Council of Türkiye (TÜBİTAK) under the 2209-A University Students Research Projects Support Program (Project ID: 1919B012426269).”  Please state what role the funders took in the study. If the funders had no role, please state: "The funders had no role in study design, data collection and analysis, decision to publish, or preparation of the manuscript."  If this statement is not correct you must amend it as needed.  Please include this amended Role of Funder statement in your cover letter; we will change the online submission form on your behalf. | We thank the journal office for this comment. We have amended the funding statement to include the role of the funders. The revised statement now reads: “This research was partially funded by the Scientific and Technological Research Council of Türkiye (TÜBİTAK) under the 2209-A University Students Research Projects Support Program (Project ID: 1919B012426269). The funders had no role in study design, data collection and analysis, decision to publish, or preparation of the manuscript.” We also included this amended Role of Funder statement in the cover letter, as requested. |
| 4. Please note that funding information should not appear in any section or other areas of your manuscript. We will only publish funding information present in the Funding Statement section of the online submission form. Please remove any funding-related text from the manuscript. | We thank the journal office for this clarification. We have checked the manuscript and removed the funding-related text from the manuscript file, including the separate Funding section. The funding information and Role of Funder statement will be provided only in the Funding Statement section of the online submission form and in the cover letter, as requested. |
| 5. We note that your Data Availability Statement is currently as follows:  “All relevant data are within the manuscript and its Supporting Information files.”  Please confirm at this time whether or not your submission contains all raw data required to replicate the results of your study. Authors must share the “minimal data set” for their submission. PLOS defines the minimal data set to consist of the data required to replicate all study findings reported in the article, as well as related metadata and methods (https://journals.plos.org/plosone/s/data-availability#loc-minimal-data-set-definition).  For example, authors should submit the following data:  - The values behind the means, standard deviations and other measures reported;  - The values used to build graphs;  - The points extracted from images for analysis.  Authors do not need to submit their entire data set if only a portion of the data was used in the reported study.  If your submission does not contain these data, please either upload them as Supporting Information files or deposit them to a stable, public repository and provide us with the relevant URLs, DOIs, or accession numbers. For a list of recommended repositories, please see https://journals.plos.org/plosone/s/recommended-repositories.  If there are ethical or legal restrictions on sharing a de-identified data set, please explain them in detail (e.g., data contain potentially sensitive information, data are owned by a third-party organization, etc.) and who has imposed them (e.g., an ethics committee). Please also provide contact information for a data access committee, ethics committee, or other institutional body to which data requests may be sent. If data are owned by a third party, please indicate how others may request data access. | We thank the journal office for this comment. We confirm that the current Data Availability Statement is correct. The submission includes the dataset used to conduct the analyses reported in the manuscript, and this Supporting Information file contains the minimal dataset required to replicate the study findings, including the values underlying the descriptive statistics, group comparisons, regression analyses, and figures. Therefore, all relevant data are available within the manuscript and its Supporting Information files. |
| 6. Please amend the manuscript submission data (via Edit Submission) to include author Dr. Zeynep Begüm KALYONCU ATASOY. | We thank the journal office for this comment. We confirm that Dr. Zeynep Begüm KALYONCU ATASOY is the corresponding author and submitting author, and she is completing the amendments through the submission system. We have reviewed the manuscript submission data via Edit Submission and amended/confirmed the author information as requested. |
| 7. Your ethics statement should only appear in the Methods section of your manuscript. If your ethics statement is written in any section besides the Methods, please move it to the Methods section and delete it from any other section. Please ensure that your ethics statement is included in your manuscript, as the ethics statement entered into the online submission form will not be published alongside your manuscript. | We thank the journal office for this clarification. We have revised the manuscript so that the ethics statement appears only in the Methods section. The duplicate ethics statement previously placed near the end of the manuscript before the References has been removed. The Methods section now includes the ethics approval information and participant consent details, as requested. |
| 8. If the reviewer comments include a recommendation to cite specific previously published works, please review and evaluate these publications to determine whether they are relevant and should be cited. There is no requirement to cite these works unless the editor has indicated otherwise. | We thank the journal office for this clarification. We reviewed the editor and reviewer comments and did not identify any recommendation to cite specific previously published works. Therefore, no additional references were added in response to this item. However, we reviewed the reference list for consistency with PLOS ONE style, as noted above. |
| **Reviewer 1** **Comments and Suggestions for Author** | **Author’s response** |
| 1. Age group of study participants? | We thank Reviewer 1 for this comment. We have clarified the age distribution of the study participants in the revised manuscript. Participants were adults aged 18 years or older, and age was presented both as a continuous variable and as descriptive age groups in Table 1. Specifically, participants were categorized as <25 years, 25–34 years, 35–44 years, and ≥45 years. This revision provides a clearer overview of the age profile of the study sample. |
| 2. Well, study seems pretty interesting and interactive. I think authors might have lacked the proper layout/study design of this behavioral study. | We thank Reviewer 1 for this comment and for noting that the study topic is interesting. In response to the concern regarding the layout and study design of this behavioral study, we revised the manuscript to improve the clarity of the study rationale, conceptual framing, and overall presentation. In particular, the Introduction now clarifies that air fryer ownership was conceptualized not as a direct nutritional exposure or inherently health-promoting technology, but as a household-level marker of contemporary kitchen technology adoption. We also strengthened the theoretical rationale linking technology ownership, household task sharing, cooking and food preparation skills, and Mediterranean diet adherence as interrelated components of domestic food practices. In addition, we revised the study aim to clarify that the purpose was to situate air fryer ownership within broader household food-practice patterns rather than to evaluate the appliance as a causal determinant of diet quality, skills, or household labour division. To further improve the layout and readability of the manuscript, we also added a workflow figure and a regression coefficient plot. |
| 3. To my surprise there is no figure in the whole research article. | We thank Reviewer 1 for this observation. In response, we have added visual material to the revised manuscript to improve clarity and readability. Specifically, we included a study flow diagram summarizing the study design, eligibility criteria, final analytic sample, air fryer ownership groups, and main survey measures. We also added regression coefficient plots to visually present the adjusted associations from the robust regression models for Mediterranean diet adherence, cooking skills, and food preparation skills. These figures complement the tabular presentation of the findings and are intended to improve the accessibility and interpretability of the methodological and analytical components of the study. |
| 4. Comparative Figures may be more impactful and easy to understand along with a few tables. | We thank Reviewer 1 for this suggestion. We agree that comparative visual presentation can make key findings easier to follow alongside the tables. In response, we added regression coefficient plots to visually summarize and compare the adjusted associations observed in the robust regression models. These figures present the direction, magnitude, and 95% confidence intervals of the associations for Mediterranean diet adherence, cooking skills, and food preparation skills. We also added a study flow diagram to improve the overall presentation of the study design, eligibility criteria, participant groups, and survey measures. These additions complement the tabular results and improve the readability and interpretability of the manuscript. |
| 5. Tables contain more detailed statistical data parameters, which I think are no more required in modern day article analysis. Yes, you do need a significance statement or decision making. | We thank Reviewer 1 for this comment. We understand this suggestion as indicating that the tables should be clearer and easier to interpret, particularly for identifying the direction and significance of the findings. In response, we reviewed the tabular presentation of the results and revised the tables to improve readability, consistency, and interpretability. In particular, Table 3 was revised to clarify that scale outcomes are presented as median (minimum–maximum), whereas categorical MEDAS outcomes are presented as n (%). We also revised table footnotes to clarify the statistical tests and notation used. For the regression tables, we retained the main statistical parameters, including coefficients, standard errors, confidence intervals, p-values, and VIF values, to preserve transparency and reproducibility of the multivariable analyses. To make the main findings easier to interpret visually, we also added regression coefficient plots summarizing the direction, magnitude, and 95% confidence intervals of the adjusted associations. These figures complement the tabular presentation and provide a clearer visual summary of the significance and direction of the findings. |
| 6. What authors are trying to evaluate from this study? Air fryer ownership effect on the behavior of users and their diet pattern? Well, if you are focusing on air fryer, then you should specifically get data for affordability of getting it? If users are unable to buy one, what are the factors behind it? You are studying a specific location, please specify that circumference, it should not be like study in Study. Is 384 nos. is the true representative of the subject population? What is meant by the air fryer ownership? Did authors check that to which individual the air fryer was registered? OR who purchased it? If both partners shared it then what is the criteria? | We thank Reviewer 1 for this detailed comment. We have revised the manuscript to clarify the aim and conceptual framing of the study. The study did not aim to evaluate the causal effect of air fryer ownership on users’ behavior or dietary patterns. Rather, air fryer ownership was conceptualized as a household-level marker of contemporary kitchen technology adoption and convenience-oriented food practices, and the study examined whether ownership was associated with household task sharing, cooking and food preparation skills, and Mediterranean diet adherence.  Regarding affordability, we agree that purchasing capacity is relevant to air fryer ownership. Although the questionnaire did not ask non-owners why they did not own an air fryer, perceived income status was assessed and included in the descriptive comparisons and regression models. In the revised Introduction and Discussion, we clarified that air fryer ownership may reflect socioeconomic positioning and purchasing capacity, alongside interest in cooking technologies and household food-practice patterns.  We also clarified the study setting and generalizability. The study was conducted in Istanbul, Türkiye, and the sample size of 384 was calculated as the minimum required sample size using finite population correction based on Turkish Statistical Institute marriage statistics for Istanbul. However, because snowball sampling was used, the sample should not be considered statistically representative of all cohabiting adults in Istanbul or Türkiye. This limitation has been made more explicit in the Methods, limitations, and Conclusion sections.  Finally, we clarified the meaning of air fryer ownership. In this study, air fryer ownership referred to self-reported household ownership of an air fryer, rather than legal registration, individual purchase, or exclusive use by one household member. Because the study focused on cohabiting adults and household food-related practices, ownership was treated as a household-level variable. We acknowledge that the purchaser, primary user, and reasons for non-ownership were not assessed, and this has been considered when interpreting the findings. |
| 7. Moreover, it is evident that women are better cook then men and they have better esthetic sense than men too. | We thank Reviewer 1 for this comment. We agree that sex differences in cooking and food preparation skills require careful interpretation. However, we do not interpret these differences as reflecting inherent ability or aesthetic sense. In the revised manuscript, we frame the observed differences in relation to gendered domestic labour, socialization, household role expectations, and unequal responsibility for food-related work. We also strengthened the Discussion to clarify that women’s higher cooking and food preparation skill scores in this sample likely reflect socially patterned exposure to household foodwork rather than innate differences. This interpretation is supported by literature on unpaid domestic labour, time use, and cognitive labour in household food practices. |
| 8. Education should be mentioned numerically like masters or bachelors or primary education etc. | We thank Reviewer 1 for this suggestion. In response, we added categorical education information to the Results section to complement the existing presentation of total years of education. The revised text now reports that categorical education level differed by air fryer ownership and that higher education was more frequent among owners than non-owners. We also added the overall distribution of the two largest education categories, noting that 51.3% of participants had higher education and 26.8% had high school education. This revision provides a clearer descriptive profile of participants’ educational background while retaining total years of education in Table 1 for statistical comparison. |
| 9. Today is the age of technology, every recipe is available on net. So, anyone can check and follow it from there to cook anything. Many more questions are discussable. | We thank Reviewer 1 for this comment. We agree that digital technologies and online recipes have made cooking information widely accessible. However, access to recipes does not necessarily mean that cooking and food-related responsibilities are equally distributed within households. Therefore, the present study focused not only on access to cooking-related resources or technologies, but also on the household organization of food-related tasks, cooking and food preparation skills, and Mediterranean diet adherence. In the revised Discussion, we added a brief statement acknowledging that the widespread availability of online recipes and cooking information may reduce informational barriers to cooking, but does not necessarily alter who assumes responsibility for planning, preparing, and coordinating meals within the household. This addition clarifies why household organization and gendered responsibility remain relevant despite the widespread availability of digital cooking resources. |
| I am afraid about the feasibility or suitability of the paper for your journal and I don’t recommend it for publication please. | We thank Reviewer 1 for the overall assessment. We have carefully considered all comments and have substantially revised the manuscript to improve clarity, conceptual framing, presentation, and interpretability. The revised manuscript now includes clearer age and education descriptions, improved table formatting and footnotes, additional visual material, more explicit discussion of sampling and generalizability, stronger non-causal wording appropriate for a cross-sectional design, and a clearer explanation of air fryer ownership as a household-level marker rather than a causal exposure. We hope that these revisions address the reviewer’s concerns and improve the suitability of the manuscript for publication in PLOS ONE. |
| **Reviewer 2** **Comments and Suggestions for Author** | **Author’s response** |
| General overview  This article is a well thought-out and excellent presentation. Although with minor observations as below.. | We thank Reviewer 2 for the positive assessment of our manuscript and for recognizing the policy relevance of this work. We appreciate the constructive comments provided and have revised the manuscript accordingly, as detailed in our point-by-point responses. |
| 1. Table 3 on line 226, (Within-group comparisons of scale scores by sex among air fryer owners and non-owners), there are 3 categories there;  a. Cooking skills  b. Food Preparation Skills  c. Mediterranean Diet Adherence Screener.  Referencing the fact that one respondent was recruited per household and N=384; 50% women; 192 participants (50%) reported owning an air fryer and the other 50 % does not; the figures reported in the Table does not add up to 384.  For category a (Cooking skills), total for ownership+ was 291 and ownership- was 272.  Total for category b (Food Preparation Skills) was 398 (ownership+) and ownership- was 385.5  Category c (Mediterranean Diet Adherence Screener), was in line (ownership+ 194 and ownership- 190).  a. How do you account for the discrepancies in the reported figures?  b. For Cooking skills, does it mean that all the participants did not respond in the category? And, under Food Preparation skills, how do you justify 398 and 385.5 respondents? (Cross check your figures again). | We thank Reviewer 2 for this important comment. We carefully rechecked the values reported in Table 3 and confirmed that there was no calculation error. However, we acknowledge that the previous presentation of the table may have created misunderstanding on our part by not making sufficiently clear that the values for cooking skills, food preparation skills, and MEDAS total score were scale scores presented as median (minimum–maximum), not numbers of participants. Therefore, these values were not expected to add up to the total sample size of 384.  To prevent this misunderstanding, we revised Table 3 substantially. The table title, row labels, and footnote were amended to clearly indicate that cooking skills, food preparation skills, and MEDAS total values are scale scores presented as median (minimum–maximum). We also separated “MEDAS Total Score” from “MEDAS Categories” and clarified that MEDAS categories are presented as n (%). Thus, only the categorical MEDAS rows represent participant counts and percentages, whereas the cooking and food preparation skill rows represent scale scores. We believe these revisions make the table clearer and prevent scale scores from being misinterpreted as participant numbers. |
| 2. On line 435, 441, 445, 458, 461, 466, 480, 483, 498, 503, 511, 514 and 527 under references, et. al is stated. Can’t the full referencing be stated? Irrespective of the referencing style of the publishing house, the full names of the authors should be stated. | We thank Reviewer 2 for this comment. We reviewed the reference list and revised the entries to ensure consistency with PLOS ONE reference style. Specifically, we replaced inconsistent uses of “et al.” with the full author list where required and checked the reference list for consistency in author names, journal titles, year, volume, issue, and page or article numbers. |
