# Supplementary material for: Air fryer ownership, household food-related task sharing, cooking skills, and mediterranean diet adherence among cohabiting adults in Türkiye: A cross-sectional study
Source: PLoS One. 2026 Jul 8;21(7):e0352090. doi: 10.1371/journal.pone.0352090 (PMC13345407; doi:10.1371/journal.pone.0352090)
Supplement: Peer review history

# Peer review: Decision Letter 0

**DOI:** 10.1371/journal.pone.0352090.r001

**Authors:** Nour Elsahoryi

29 Apr 2026

PONE-D-26-12396

Air fryer ownership, household food-related task sharing, cooking skills, and mediterranean diet adherence among cohabiting adults in Türkiye: a cross-sectional study

PLOS One

Dear Dr, Kalyoncu Atasoy,

Thank you for submitting your manuscript to PLOS ONE. After careful consideration, we feel that it has merit but does not fully meet PLOS ONE’s publication criteria as it currently stands. Therefore, we invite you to submit a revised version of the manuscript that addresses the points raised during the review process.

Please submit your revised manuscript by Jun 13 2026 11:59PM. If you will need more time than this to complete your revisions, please reply to this message or contact the journal office at plosone@plos.org. When you're ready to submit your revision, log on to https://www.editorialmanager.com/pone/ and select the 'Submissions Needing Revision' folder to locate your manuscript file.

Please include the following items when submitting your revised manuscript:

- A letter that responds to each point raised by the academic editor and reviewer(s). You should upload this letter as a separate file labeled 'Response to Reviewers'.
- A marked-up copy of your manuscript that highlights changes made to the original version. You should upload this as a separate file labeled 'Revised Manuscript with Track Changes'.
- An unmarked version of your revised paper without tracked changes. You should upload this as a separate file labeled 'Manuscript'.

If you would like to make changes to your financial disclosure, please include your updated statement in your cover letter. Guidelines for resubmitting your figure files are available below the reviewer comments at the end of this letter.

If applicable, we recommend that you deposit your laboratory protocols in protocols.io to enhance the reproducibility of your results. Protocols.io assigns your protocol its own identifier (DOI) so that it can be cited independently in the future. For instructions see: https://journals.plos.org/plosone/s/submission-guidelines#loc-laboratory-protocols. Additionally, PLOS ONE offers an option for publishing peer-reviewed Lab Protocol articles, which describe protocols hosted on protocols.io. Read more information on sharing protocols at https://plos.org/protocols?utm_medium=editorial-email&utm_source=authorletters&utm_campaign=protocols.

As the corresponding author, your ORCID iD is verified in the submission system and will appear in the published article. PLOS supports the use of ORCID, and we encourage all coauthors to register for an ORCID iD and use it as well. Please encourage your coauthors to verify their ORCID iD within the submission system before final acceptance, as unverified ORCID iDs will not appear in the published article. *Only*  the individual author can complete the verification step; PLOS staff *cannot*  verify ORCID iDs on behalf of authors.

We look forward to receiving your revised manuscript.

Kind regards,

Nour Amin Elsahoryi, pHD

Academic Editor

PLOS One

**Journal Requirements:**

When submitting your revision, we need you to address these additional requirements.

1. Please ensure that your manuscript meets PLOS ONE's style requirements, including those for file naming. The PLOS ONE style templates can be found at

https://journals.plos.org/plosone/s/file?id=wjVg/PLOSOne_formatting_sample_main_body.pdf and

https://journals.plos.org/plosone/s/file?id=ba62/PLOSOne_formatting_sample_title_authors_affiliations.pdf

2. Please provide additional details regarding participant consent. In the ethics statement in the Methods and online submission information, please ensure that you have specified (1) whether consent was informed and (2) what type you obtained (for instance, written or verbal, and if verbal, how it was documented and witnessed). If your study included minors, state whether you obtained consent from parents or guardians. If the need for consent was waived by the ethics committee, please include this information.

If you are reporting a retrospective study of medical records or archived samples, please ensure that you have discussed whether all data were fully anonymized before you accessed them and/or whether the IRB or ethics committee waived the requirement for informed consent. If patients provided informed written consent to have data from their medical records used in research, please include this information.

3. Thank you for stating the following financial disclosure:

“This research was partially funded by the Scientific and Technological Research Council of Türkiye (TÜBİTAK) under the 2209-A University Students Research Projects Support Program (Project ID: 1919B012426269).”

Please state what role the funders took in the study.  If the funders had no role, please state: "The funders had no role in study design, data collection and analysis, decision to publish, or preparation of the manuscript."

If this statement is not correct you must amend it as needed.

Please include this amended Role of Funder statement in your cover letter; we will change the online submission form on your behalf.

4. Please note that funding information should not appear in any section or other areas of your manuscript. We will only publish funding information present in the Funding Statement section of the online submission form. Please remove any funding-related text from the manuscript.

5. We note that your Data Availability Statement is currently as follows:

“All relevant data are within the manuscript and its Supporting Information files.”

Please confirm at this time whether or not your submission contains all raw data required to replicate the results of your study. Authors must share the “minimal data set” for their submission. PLOS defines the minimal data set to consist of the data required to replicate all study findings reported in the article, as well as related metadata and methods (https://journals.plos.org/plosone/s/data-availability#loc-minimal-data-set-definition).

For example, authors should submit the following data:

- The values behind the means, standard deviations and other measures reported;

- The values used to build graphs;

- The points extracted from images for analysis.

Authors do not need to submit their entire data set if only a portion of the data was used in the reported study.

If your submission does not contain these data, please either upload them as Supporting Information files or deposit them to a stable, public repository and provide us with the relevant URLs, DOIs, or accession numbers. For a list of recommended repositories, please see https://journals.plos.org/plosone/s/recommended-repositories.

If there are ethical or legal restrictions on sharing a de-identified data set, please explain them in detail (e.g., data contain potentially sensitive information, data are owned by a third-party organization, etc.) and who has imposed them (e.g., an ethics committee). Please also provide contact information for a data access committee, ethics committee, or other institutional body to which data requests may be sent. If data are owned by a third party, please indicate how others may request data access.

6. Please amend the manuscript submission data (via Edit Submission) to include author Dr. Zeynep Begüm KALYONCU ATASOY.

7. Your ethics statement should only appear in the Methods section of your manuscript. If your ethics statement is written in any section besides the Methods, please move it to the Methods section and delete it from any other section. Please ensure that your ethics statement is included in your manuscript, as the ethics statement entered into the online submission form will not be published alongside your manuscript.

8. If the reviewer comments include a recommendation to cite specific previously published works, please review and evaluate these publications to determine whether they are relevant and should be cited. There is no requirement to cite these works unless the editor has indicated otherwise.

**Additional Editor Comments:**

This manuscript addresses an interesting and underexplored topic by integrating household technology use with food-related behaviors and dietary outcomes. The study is well motivated, and the use of validated tools alongside regression modelling strengthens the analytical approach. However, several important issues should be addressed to improve clarity, rigor, and interpretability.

Major Issues

Clarity and Consistency of Data Presentation (Table 3)

Table 3 is currently difficult to interpret and may be misleading. The reported values appear inconsistent with the stated sample size (N=384), which creates confusion regarding whether the values represent counts, medians, or scale scores. This issue was also identified by another reviewer and must be addressed.

Clearly specify the unit of analysis (e.g., median [min–max], score, or n).

Ensure that totals are not misinterpreted as participant counts.

Consider restructuring the table for readability (e.g., separating subscales or presenting summary scores only).

Interpretation of Associations (Causality)

The manuscript occasionally implies directional or causal relationships (e.g., ownership influencing skills or behaviors). Given the cross-sectional design, all findings should be framed strictly as associations.

Revise wording throughout (especially Discussion) to avoid causal language.

Emphasize potential reverse causality and residual confounding.

Sampling Strategy and Generalizability

The use of snowball sampling limits representativeness and introduces selection bias.

This limitation should be more explicitly acknowledged.

Avoid generalizing findings beyond the sampled population.

Conceptual Alignment of Study Objectives

The manuscript combines multiple constructs (technology ownership, task sharing, skills, and diet adherence), but the conceptual linkage between them could be more explicitly articulated.

Clarify whether air fryer ownership is conceptualized as a proxy for lifestyle, convenience behavior, or socioeconomic positioning.

Strengthen the theoretical rationale linking these domains.

Minor Issues

Figures and Visual Presentation

Although not mandatory, the inclusion of at least one figure would improve clarity.

Suggested: conceptual framework or regression coefficient plot.

Statistical Reporting

Ensure consistent reporting format across tables (especially medians vs means).

Clarify all test statistics and notation in table footnotes.

Referencing Style

Some references use “et al.” inconsistently; ensure alignment with journal guidelines.

Language and Style

Minor grammatical and stylistic edits are needed to improve clarity and readability.

[Note: HTML markup is below. Please do not edit.]

Reviewers' comments:

Reviewer's Responses to Questions

**Comments to the Author**

1. Is the manuscript technically sound, and do the data support the conclusions?

The manuscript must describe a technically sound piece of scientific research with data that supports the conclusions. Experiments must have been conducted rigorously, with appropriate controls, replication, and sample sizes. The conclusions must be drawn appropriately based on the data presented.

Reviewer #1: No

Reviewer #2: Yes

**********

2. Has the statistical analysis been performed appropriately and rigorously?

Reviewer #1: I Don't Know

Reviewer #2: Yes

**********

3. Have the authors made all data underlying the findings in their manuscript fully available?

The PLOS Data policy requires authors to make all data underlying the findings described in their manuscript fully available without restriction, with rare exception (please refer to the Data Availability Statement in the manuscript PDF file). The data should be provided as part of the manuscript or its supporting information, or deposited to a public repository. For example, in addition to summary statistics, the data points behind means, medians and variance measures should be available. If there are restrictions on publicly sharing data—e.g. participant privacy or use of data from a third party—those must be specified.

Reviewer #1: Yes

Reviewer #2: Yes

**********

4. Is the manuscript presented in an intelligible fashion and written in standard English?

PLOS ONE does not copyedit accepted manuscripts, so the language in submitted articles must be clear, correct, and unambiguous. Any typographical or grammatical errors should be corrected at revision, so please note any specific errors here.

Reviewer #1: No

Reviewer #2: Yes

**********

5. Review Comments to the Author

Please use the space provided to explain your answers to the questions above. You may also include additional comments for the author, including concerns about dual publication, research ethics, or publication ethics. (Please upload your review as an attachment if it exceeds 20,000 characters)

Reviewer #1: Confidential Comments to Editor

1. Age group of study participants?

2. Well, study seems pretty interesting and interactive. I think authors might have lacked the proper layout/study design of this behavioral study.

3. To my surprise there is no figure in the whole research article.

4. Comparative Figures may be more impactful and easy to understand along with a few tables.

5. Tables contain more detailed statistical data parameters, which I think are no more required in modern day article analysis. Yes, you do need a significance statement or decision making.

6. What authors are trying to evaluate from this study? Air fryer ownership effect on the behavior of users and their diet pattern? Well, if you are focusing on air fryer, then you should specifically get data for affordability of getting it? If users are unable to buy one, what are the factors behind it? You are studying a specific location, please specify that circumference, it should not be like study in Study. Is 384 nos. is the true representative of the subject population? What is meant by the air fryer ownership? Did authors check that to which individual the air fryer was registered? OR who purchased it? If both partners shared it then what is the criteria?

7. Moreover, it is evident that women are better cook then men and they have better esthetic sense than men too.

8. Education should be mentioned numerically like masters or bachelors or primary education etc.

9. Today is the age of technology, every recipe is available on net. So, anyone can check and follow it from there to cook anything. Many more questions are discussable.

Reviewer’s Judgement:

I am afraid about the feasibility or suitability of the paper for your journal and I don’t recommend it for publication please.

Reviewer #2: AIR FRYER OWNERSHIP, HOUSEHOLD FOOD-RELATED TASK SHARING, COOKING SKILLS, AND MEDITERRANEAN DIET ADHERENCE AMONG COHABITING ADULTS IN TÜRKIYE: A CROSS-SECTIONAL STUDY.

General overview

This article is a well thought-out and excellent presentation. Although with minor observations as below..

Observations

1. Table 3 on line 226, (Within-group comparisons of scale scores by sex among air fryer owners and non-owners), there are 3 categories there;

a. Cooking skills

b. Food Preparation Skills

c. Mediterranean Diet Adherence Screener.

Referencing the fact that one respondent was recruited per household and N=384; 50% women; 192 participants (50%) reported owning an air fryer and the other 50 % does not; the figures reported in the Table does not add up to 384.

For category a (Cooking skills), total for ownership+ was 291 and ownership- was 272.

Total for category b (Food Preparation Skills) was 398 (ownership+) and ownership- was 385.5

Category c (Mediterranean Diet Adherence Screener), was in line (ownership+ 194 and ownership- 190).

a. How do you account for the discrepancies in the reported figures?

b. For Cooking skills, does it mean that all the participants did not respond in the category? And, under Food Preparation skills, how do you justify 398 and 385.5 respondents? (Cross check your figures again).

2. On line 435, 441, 445, 458, 461, 466, 480, 483, 498, 503, 511, 514 and 527 under references, et. al is stated. Can’t the full referencing be stated? Irrespective of the referencing style of the publishing house, the full names of the authors should be stated.

**********

6. PLOS authors have the option to publish the peer review history of their article (what does this mean?). If published, this will include your full peer review and any attached files.

If you choose “no”, your identity will remain anonymous but your review may still be made public.

**Do you want your identity to be public for this peer review?**  For information about this choice, including consent withdrawal, please see our Privacy Policy.

Reviewer #1: **Yes:**  Tauqeer Ahmad

Reviewer #2: No

**********

[NOTE: If reviewer comments were submitted as an attachment file, they will be attached to this email and accessible via the submission site. Please log into your account, locate the manuscript record, and check for the action link "View Attachments". If this link does not appear, there are no attachment files.]

To ensure your figures meet our technical requirements, please review our figure guidelines: https://journals.plos.org/plosone/s/figures

You may also use PLOS’s free figure tool, NAAS, to help you prepare publication quality figures: https://journals.plos.org/plosone/s/figures#loc-tools-for-figure-preparation.

NAAS will assess whether your figures meet our technical requirements by comparing each figure against our figure specifications.

Attachment Submitted filename: Reviewer Confidential Comments to Editor.pdf

# Author comment: Author response to Decision Letter 1

**DOI:** 10.1371/journal.pone.0352090.r002

11 May 2026

Dear Editors,

We have carefully revised the manuscript in response to the Academic Editor’s, reviewers’, and journal office comments. A detailed point-by-point response to all comments has been prepared and uploaded as a separate Response to Reviewers file. The revised manuscript includes changes to the conceptual framing, table presentation, figure presentation, statistical reporting, participant consent details, sampling/generalizability limitations, non-causal interpretation, funding information, and data availability confirmation.

Kind regards,

Zeynep Begum Kalyoncu Atasoy

Attachment Submitted filename: Response_to_Reviewers_airfryer_zbka.docx

# Editor report: Decision Letter 1

**DOI:** 10.1371/journal.pone.0352090.r003

**Authors:** Nour Elsahoryi, Nour Elsahoryi

5 Jun 2026

Air fryer ownership, household food-related task sharing, cooking skills, and mediterranean diet adherence among cohabiting adults in Türkiye: a cross-sectional study

PONE-D-26-12396R1

Dear Dr.

We’re pleased to inform you that your manuscript has been judged scientifically suitable for publication and will be formally accepted for publication once it meets all outstanding technical requirements.

Within one week, you’ll receive an e-mail detailing the required amendments. When these have been addressed, you’ll receive a formal acceptance letter and your manuscript will be scheduled for publication.

An invoice will be generated when your article is formally accepted. Please note, if your institution has a publishing partnership with PLOS and your article meets the relevant criteria, all or part of your publication costs will be covered. Please make sure your user information is up-to-date by logging into Editorial Manager at Editorial Manager® and clicking the ‘Update My Information' link at the top of the page. For questions related to billing, please contact billing support.

If your institution or institutions have a press office, please notify them about your upcoming paper to help maximize its impact. If they’ll be preparing press materials, please inform our press team as soon as possible -- no later than 48 hours after receiving the formal acceptance. Your manuscript will remain under strict press embargo until 2 pm Eastern Time on the date of publication. For more information, please contact onepress@plos.org.

Kind regards,

Nour Amin Elsahoryi, pHD

Academic Editor

PLOS One

Additional Editor Comments (optional):

Thank you for your careful revision. The manuscript has improved substantially. The main concerns regarding conceptual framing, non-causal interpretation, sampling limitations, table clarity, figures, ethics/consent, funding role, and data availability have been adequately addressed.I have no further major concerns. Please complete a final editorial check, particularly to remove any residual template text from the title page and ensure consistent formatting before publication.

Reviewers' comments:

# Editor report: Acceptance letter

**DOI:** 10.1371/journal.pone.0352090.r004

**Authors:** Nour Elsahoryi, Nour Elsahoryi

PONE-D-26-12396R1

PLOS One

Dear Dr. Begüm KALYONCU ATASOY,

I'm pleased to inform you that your manuscript has been deemed suitable for publication in PLOS One. Congratulations! Your manuscript is now being handed over to our production team.

At this stage, our production department will prepare your paper for publication. This includes ensuring the following:

* All references, tables, and figures are properly cited

* All relevant supporting information is included in the manuscript submission,

* There are no issues that prevent the paper from being properly typeset

You will receive further instructions from the production team, including instructions on how to review your proof when it is ready. Please keep in mind that we are working through a large volume of accepted articles, so please give us a few days to review your paper and let you know the next and final steps.

Lastly, if your institution or institutions have a press office, please let them know about your upcoming paper now to help maximize its impact. If they'll be preparing press materials, please inform our press team within the next 48 hours. Your manuscript will remain under strict press embargo until 2 pm Eastern Time on the date of publication. For more information, please contact onepress@plos.org.

You will receive an invoice from PLOS for your publication fee after your manuscript has reached the completed accept phase. If you receive an email requesting payment before acceptance or for any other service, this may be a phishing scheme. Learn how to identify phishing emails and protect your accounts at https://explore.plos.org/phishing.

If we can help with anything else, please email us at customercare@plos.org.

Thank you for submitting your work to PLOS ONE and supporting open access.

Kind regards,

PLOS ONE Editorial Office Staff

on behalf of

Dr. Nour Amin Elsahoryi

Academic Editor

PLOS One
